# PPARgamma-Dependent Control of Renin Expression: Molecular Mechanisms and Pathophysiological Relevance

**DOI:** 10.1155/2013/451016

**Published:** 2013-10-30

**Authors:** Vladimir T. Todorov

**Affiliations:** Experimental Nephrology, Division of Nephrology, Department of Medicine III, University Hospital Carl Gustav Carus, TU Dresden, 01307 Dresden, Germany

## Abstract

During the last years accumulating evidence demonstrated that the nuclear receptor peroxisome proliferator-activated receptor-gamma (PPARgamma) regulates the expression of renin gene and thus the overall renin production. This review summarizes the current knowledge of the transcriptional control of the renin gene by PPARgamma received from variety of models ranging from cell culture to transgenic animals. The molecular mechanisms of the PPARgamma action on renin are particularly interesting because they are featured by two newly described characteristics: one of them is the recently identified PPARgamma target sequence Pal3 which is specific for the human renin gene and mediates exceptionally high sensitivity to transactivation; the other is the potentiating effect of PPARgamma on the cAMP signaling in the renin-producing cells. Furthermore, I discuss the need for generating of additional transgenic animal models which are more appropriate with regard to the role of the PPARgamma-dependent regulation of the renin gene expression in human diseases such as arterial hypertension and metabolic syndrome.

## 1. Introduction

Renin is aspartyl protease produced by the juxtaglomerular (JG) cells in the afferent arterioles of the kidney. It is the limiting enzyme in renin-angiotensin system (RAS), which plays crucial role in the control of blood pressure and salt excretion. The renin production is tightly regulated at the transcriptional level. Although the active renin is released into the circulation through regulated exocytosis, chronic (patho)physiological cues influencing the renin production (e.g., alterations in the salt intake, changes in the blood pressure, angiotensin II blockade, etc.) always induce parallel changes in the plasma renin concentration (PRC) and the renin mRNA levels in the JG cells [1]. Therefore, the control of the gene transcription is the decisive step in the overall regulation of the renin production. The *cis*-acting regulatory sequences of the renin gene are located in the 5'-flanking promoter. The renin promoter has two evolutionary conserved regulatory regions: the proximal promoter which lies immediately upstream of the transcription starting site and the distal (or kidney) enhancer which consists of approximately 240 bp located at around −2.6 kb in the mouse and −12 kb in the human renin gene [2]. Many transcription factors acting through recognition sequences in the proximal promoter or the kidney enhancer are involved in the regulation of the renin gene [1]. Most of the experimental data on the function of these transcription factors was obtained from cells culture setups. Currently, in vivo models are intensively used to decipher the transcriptional control of the renin gene. Although some of the in vivo findings do not confirm the earlier in vitro results (which may also reflect species-specific differences), the overall data on the regulation of the renin expression fits good together and provides a comprehensive insight into the regulatory mechanisms engaged.

**Figure 1:**
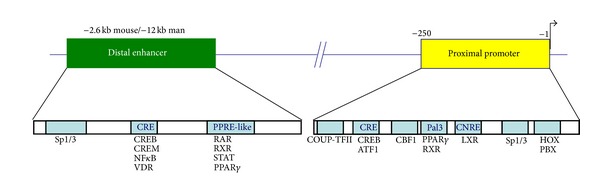
Map of the 5′-flanking regulatory promoter elements of the renin gene and the interacting transcription factors mentioned in the text.

The transcription factors driving the renin gene could be divided into two groups based on their functional role and their promoter interaction site (Figure 1). The first group includes transcriptional regulators which control the basal expression of the renin gene. Most (but not all) of them interact with the proximal renin promoter. This group includes members of CREB/ATF, nuclear receptor, CBF/HOX/PBX, and Sp/KLF transcription factor families [3–5]. It is believed that the concerted action of these proteins is responsible for the developmental control of the renin gene, which is highlighted by a unique temporal and site-specific expression pattern throughout the developing kidney vasculature. The second group consists of factors which regulate the renin transcription in response to homeostatic or pathophysiological signals. Important representatives of this group are CREB, nuclear receptors (such as LXR, RAR/RXR, VDR, COUP-TFII, and PPARgamma), STATs, and NFkappaB [6–12]. Notably, CREB and the nuclear receptors could both bind to the distal enhancer and the proximal promoter, while STATs and NFkappaB interact only with the enhancer element. Based on this binding pattern, it could be assumed that CREB and the nuclear receptors are particularly important for the control of the renin gene. It is now accepted that CREB plays central role in the regulation of the renin expression [13]. CREB is the major transcriptional effector of the cAMP/PKA signaling cascade which in turn is assumed to be the most important intracellular mechanism driving the renin synthesis [1]. As to the nuclear receptors, it appears that various members of the family participate in the control of both basal and regulated renin gene transcription. One of their modes of action is to modulate the effect of the cAMP/PKA pathway on the renin gene [10, 12, 14, 15]. However, the overall mechanisms through which the nuclear receptors influence the renin transcription (although still not completely characterized) are much more complex. They include various input signals and interactions within the nuclear receptor superfamily as well as with multiple renin promoter recognition sites. This review focuses on the role of the nuclear receptor PPARgamma in the regulation of the renin gene expression because PPARgamma is exemplary for the multimodal molecular actions of the nuclear receptors on the renin transcription. In addition, the PPARgamma-dependent transactivation of the renin gene could be one of the mechanisms inducing hypertension in obesity and its accompanying diseases, diabetes mellitus type 2 (DM2) and metabolic syndrome (MetS).

## 2. Transcriptional Mechanisms

PPARgamma belongs to a family of nuclear receptors which also includes PPARalpha and PPARdelta/beta. The PPARs are class I ligand-activated nuclear receptors (termed NR1C1 to C3 according to the unified nuclear receptor nomenclature) and are generally induced by fatty acids. They bind to DNA as heterodimers with RXR to a core recognition sequence known as PPRE (PPAR response element) or DR1 since it represents a direct repeat with 1 bp spacer of the consensus nuclear receptor binding site 5′-A(G)GGTCA-3′ (termed hormone response element (HRE)). Although the PPARs were originally described to regulate the lipid metabolism, it is now known that they are ubiquitously expressed and involved in multiple aspects of the control of whole body homeostasis [16, 17]. 

PPARgamma was originally identified to stimulate the renin production during screening of the effect of free fatty acids on renin in an attempt to identify mechanisms responsible for the development of arterial hypertension during obesity [18]. It was then confirmed that pharmacological PPARgamma agonists (thiazolidinediones (TZDs)) also increase the renin synthesis. The early experiments showed that the effect of PPARgamma is transcriptional because it could be abolished by general inhibitors of transcription thus excluding an effect on renin mRNA stability [18]. PPARgamma also stimulated the renin release from cultured native JG cells after at least 16 hours of agonist treatment indicating that the increase is secondary to the stimulated renin gene expression (V. T. Todorov, unpublished results). While this initial data fitted to the knowledge of the cellular mechanism of the PPARgamma action, the further findings added new insights to the ruling paradigm. We identified a PPARgamma binding site in the proximal promoter of the human renin gene, which, however, was not a PPRE/DR1 sequence. Instead it represented a palindrome with 3 bp spacer and therefore was termed Pal3. Pal3 was originally discovered in random site selection assay with in vitro translated proteins and was featured by its ability to bind PPARgamma not only as heterodimer with RXRalpha but also as a homodimer [19]. The dual binding specificity of PPARgamma was attributed to the D-box in the DNA-binding and dimerization (DBD) domain which is composed of three amino acids, while almost all nuclear receptors except the PPARs have D-box with five amino acids [19]. We provided the first evidence that PPARgamma binds to the Pal3 sequence of the human renin gene both as homodimer and as heterodimer with RXRalpha in a cell-based system [18, 20]. Surprisingly, PPARgamma induced stronger transcriptional activation through the Pal3 site as compared to the consensus PPRE/DR1 element upon agonist treatment [18]. The higher rate of transactivation through Pal3 seems to be a consequence of the binding of PPARgamma homodimers. The PPARgamma homodimer bound to Pal3 provides an additional ligand-binding site for PPARgamma agonists within the transcription factor complex as compared to the PPARgamma/RXRalpha heterodimer. This should result in more efficient recruitment of coactivators and thus in stronger transactivation of the target gene upon induction with PPARgamma agonists. Since PPARgamma binds to Pal3 with higher affinity as homodimer than as heterodimer, it could be predicted that the Pal3 sequence would be completely functional even at minimal cellular expression level of PPARgamma [19]. Indeed, the human renin Pal3 transmitted maximal PPARgamma-induced transactivation when PPARgamma was knocked down by sequence-specific siRNA, although the basal renin transcription was diminished [20]. These findings evidenced that the main function of Pal3 might be the control of PPARgamma-regulated genes in cell types with low PPARgamma expression. Although additional Pal3-driven genes were already identified (see below), the global role of Pal3 in the transcriptional control awaits further investigation. Genomewide studies demonstrated that 25% of the PPARgamma target genes (about 1400 to 3300 according to the different studies) do not possess PPRE/DR1 motif suggesting that at least 300 possible Pal3-regulated genes may exist [21, 22].

We also found that the combination of the dual binding pattern of PPARgamma with the promiscuous heterodimerization of RXR with nuclear receptors at other HRE-binding sites could contribute to the preserved binding of PPARgamma homodimers to Pal3 at low cellular expression of PPARgamma [20]. This provides additional explanation for the consistent maximal transactivation mediated by the human renin Pal3 (see above). Albeit such model seems counterintuitive, the experimental data clearly demonstrated that the binding of PPARgamma homodimers rather increases when PPARgamma is knocked down, while the overall transcription factor binding to the human renin Pal3 decreases essentially due to the impaired binding of the PPARgamma/RXRalpha heterodimers. Importantly, the renin-producing cells are devoid of estrogen receptors which bind to Pal3 sites and could have competed the binding of the PPARgamma homodimers [20].

Next to the classical ligand-dependent activation, posttranslational modification by the small ubiquitin-like modifier (SUMO) was found to modulate the PPARgamma function (for comprehensive review, see [23]). Noteworthy, the SUMOylation is a central step in the molecular mechanism of the PPARgamma anti-inflammatory action [24]. However, the impact of PPARgamma SUMOylation on the Pal3-regulated gene expression has yet to be reported.

The mouse orthologue of the human renin Pal3 turned out to be functionally silent in spite of the high sequence similarity. Consequently a quest for the PPARgamma-binding site in the mouse renin gene started. Such sequence proved to be the direct repeat HRE element in the kidney enhancer which had already been reported to mediate the upregulation of the renin expression by vitamin A [9, 20]. This motif differs from the consensus PPRE/DR1 site in a way that it has a longer spacer and therefore was also termed PPRE-like. Altogether the data from cell culture studies demonstrated that PPARgamma influences the renin transcription in a species-dependent manner and implies that the human renin gene is more sensitive to PPARgamma than the mouse renin gene. To examine the impact of PPARgamma on renin in vivo, our group generated double-transgenic mice with disrupted PPARgamma locus in the renin-producing cells. The selective PPARgamma knockout resulted in increased mouse renin expression [25]. Although this finding appears paradoxical, it was in fact expected. We and others observed that the PPARgamma deficiency increases the baseline PPRE-driven transcription [17, 20]. One of the underlying molecular mechanisms of this effect is the increased binding of nuclear receptors other than PPARgamma because PPRE/DR is generally targeted by the nonsteroidal nuclear receptors of classes I and II. The second mechanism is the impaired recruitment of transcriptional corepressors because PPARgamma resides at PPRE/DR complexed with NCoR (nuclear co-repressor) or SMRT (silencing mediator of retinoid and thyroid receptors) in the absence of ligand. In such a way, the PPARgamma deficiency and ligand-dependent activation could yield similar effects on gene transcription. Hence, the findings in an animal model confirmed the predicted effect of PPARgamma on the mouse renin gene and provided first evidence that the PPARgamma-dependent regulation of the renin transcription is relevant in vivo.

## 3. Interaction with the cAMP/PKA Pathway

A large board of data from cell culture and in vivo studies unequivocally demonstrates that the cAMP/PKA/CREB pathway is central for the control of the renin gene transcription and the overall renin production (reviewed in [1]). cAMP mediates the stimulation of the renin expression by catecholamines/sympathetic activation and prostaglandins. In addition, the cAMP/PKA signaling determines the basal transcription rate of the renin gene. The nuclear receptors interact with the cAMP/PKA cascade in the regulation of the renin gene in distinct modes. Thus, the liver X receptor (LXR) is a direct target of PKA [10]. LXR binds to a proximal promoter sequence termed CNRE (cAMP and negative regulatory element) to stimulate the renin expression in response to cAMP. On the other hand, the vitamin D_3_ receptor (VDR) heterodimerizes with CREB bound to the renin enhancer CRE (cAMP response element) to repress the renin transcription [12]. Furthermore, COUP-TFII was found to be necessary for the function of the renin gene proximal promoter CRE [15]. PPARgamma also interacts with the cAMP signaling in the renin-producing cells through a unique mechanism. The deciphering of this mechanism began with the in vitro observation that the cAMP-induced renin expression is drastically potentiated in the presence of PPARgamma agonists to peak over 100-fold (!) above the baseline [14]. The potentiation was paralleled by corresponding changes in the agonist-stimulated cAMP generation (but not in cAMP degradation) and was dependent on intact transcription. Therefore, the expression of adenylate cyclases (ACs) which are the cAMP generating enzymes was screened leading to the identification of AC6 as a PPARgamma-inducible gene. AC6 was the next PPARgamma target gene identified to be regulated by a conserved Pal3 sequence. The AC6 Pal3 sequence in its native genomic context in the kidney was found to be occupied by PPARgamma suggesting that this mechanism is functional in vivo [14].

**Figure 2:**
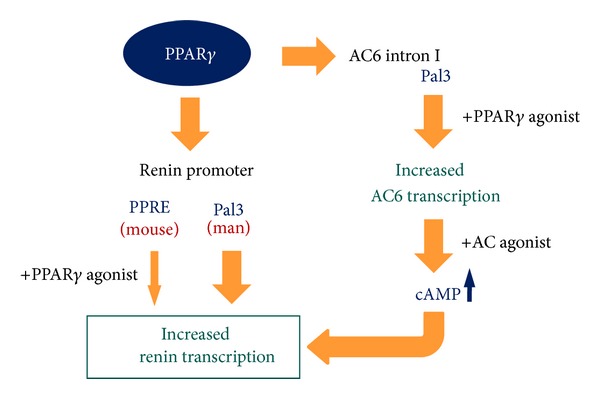
Cellular mechanisms of the PPARgamma action on the renin gene transcription.

In summary, PPARgamma appeared to stimulate the renin expression via a dual mechanism: directly, through the PPARgamma-binding sequences in the renin promoter; and indirectly, by amplifying the agonist-induced cAMP production through increased AC6 transcription (Figure 2).

## 4. Clinical Relevance and Outlook

PPARgamma is considered as the key molecule in the pathogenesis of MetS. This syndrome includes obesity accompanied by hyperlipidemia, insulin resistance/diabetes mellitus type 2, and arterial hypertension [16]. Accordingly, TZDs could be used for the treatment of insulin resistance in MetS [26]. PPARgamma is expressed in the cardiovascular system and is involved in the regulation of blood pressure. However, whether PPARgamma acts, pro- or antihypertensively is still unclear. Animal experiments showed that endothelial PPARgamma favors vasodilation and counteracts vasoconstriction [17, 27, 28]. On the other hand, studies with TZDs in endothelial cells do not adequately demonstrate that the effects of the agonists are mediated by PPARgamma [29, 30]. This issue is particularly important because TZDs may induce relaxation of vascular smooth muscle cells (VSMC) in a PPARgamma-independent manner [31, 32]. Furthermore, transgenic animals generated to selectively antagonize PPARgamma in VSMC were either hypo- or hypertensive [33–35]. At least in part these discrepant findings could be explained with the different cell specificity of the promoter constructs used to target VSMC. The transgenic strategies also involved overexpression of dominant-negative PPARgamma mutants or deletion of the protein. Our findings suggest that these two approaches should have divergent effects on Pal3 and PPRE/DR1-controlled genes, thus further complicating the interpretation of the data. It should also be noted that species-specific PPARgamma actions have to be considered before applying the knowledge from animal experiments to man [18, 20, 36]. Notwithstanding, TZDs were recommended to be suspended from the European Union market due to “an increased cardiovascular risk” (EMA/585784/2010, Press Release from 23.10.2010). In addition, a phase III trial testing the effect of a dual PPARalpha/gamma agonist on cardiovascular disease in type 2 diabetes patients has been recently halted because of “safety signals and lack of efficacy” (Roche Media Release, Basel, 10. July 2013). The latter was particularly disappointing because an initial short-term study reported promising effects of the drug on the circulation [37]. Thus, the current knowledge implies that the pharmacological activation of PPARgamma either alone or in combination with PPARalpha is not beneficial for the cardiovascular system.

**Figure 3:**
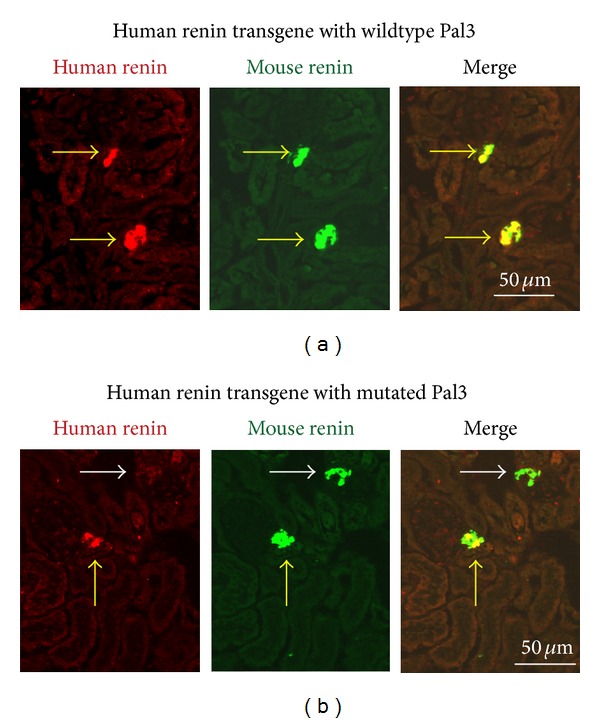
Renin expression in kidneys of mice carrying wildtype or mutated Pal3 sequence in a human renin transgene. (a) Kidney sections from mice with wildtype Pal3 stained for mouse and human renin. The human renin is expressed as expected together with the mouse renin in the JG cells (yellow arrows). (b) Kidney sections from mice with mutated Pal3 stained for mouse and human renin. The human renin is expressed again together with the mouse renin in the JG cells (yellow arrow), but there are also JG cells negative for human renin (white arrow) demonstrating that the human renin expression is diminished.

The plasma renin concentration is increased in obesity and contributes significantly to cardiovascular and renal complications (hypertension, atherosclerosis, coronary heart disease, and chronic kidney disease) [38]. Since PPARgamma is activated during obesity [39] and since it stimulates the renin gene expression [14, 18, 20], the intriguing possibility exists that the activated PPARgamma increases the renin production in MetS patients, thus acting prohypertensively. However, the experimental data in support of this hypothesis is still scarce. One major obstacle is the lack of an appropriate animal model taking into consideration that mouse and rat renin genes are likely to be less sensitive to PPARgamma than the human renin gene. In support of this assumption, renin was upregulated in healthy man but not in mice treated with TZDs ([40], V. T. Todorov, unpublished results). We treated the renin cell PPARgamma-deficient mice and their wildtype control animals with high-fat diet for up to 10 weeks. Only transient increase (at the end of the first week of treatment) of renin expression and arterial pressure in the wildtypes was observed suggesting that the conditional PPARgamma knockout mice have also limited relevance as a model for studying the hypertension in human MetS. To provide a better experimental model, our group used a transgene coplacement strategy to generate two transgenic mouse lines each of which carries the complete human renin gene inserted at one and the same genome locus. Of note the mouse and human PPARgamma molecules are identical in their DBD domains, thus excluding potential species-specific differences in the PPARgamma binding to the human renin gene in the transgenic animals. One of the lines carries the wildtype human renin gene, while the other line has a mutated Pal3 sequence in the human renin gene. This approach allows the precise comparison of the regulation of the human renin gene by PPARgamma in the presence and in the absence of the Pal3 site in vivo. The first studies revealed that the human renin expression was lower in the mice with the mutated Pal3 sequence (Figure 3). This finding corroborated the cell culture experiments where renin transcription decreased if Pal3 was mutated or if PPARgamma was knocked down [18, 20]. We are currently studying the effect of high-fat diet on the two transgenic lines to test whether Pal3 is necessary for the upregulation of the renin expression in this model of obesity. RAS in the two human renin lines will be further humanized for angiotensinogen because the human renin does not react with the mouse angiotensinogen. At the same time mouse angiotensinogen and renin will be knocked out. This strategy aims to functionally replace the endogenous mouse RAS by its human counterpart. Thus, the coplacement technology is a promising tool for the generation of a relevant animal model to decipher the role of PPARgamma and Pal3 in the stimulation of the renin production and the development of arterial hypertension during MetS.
